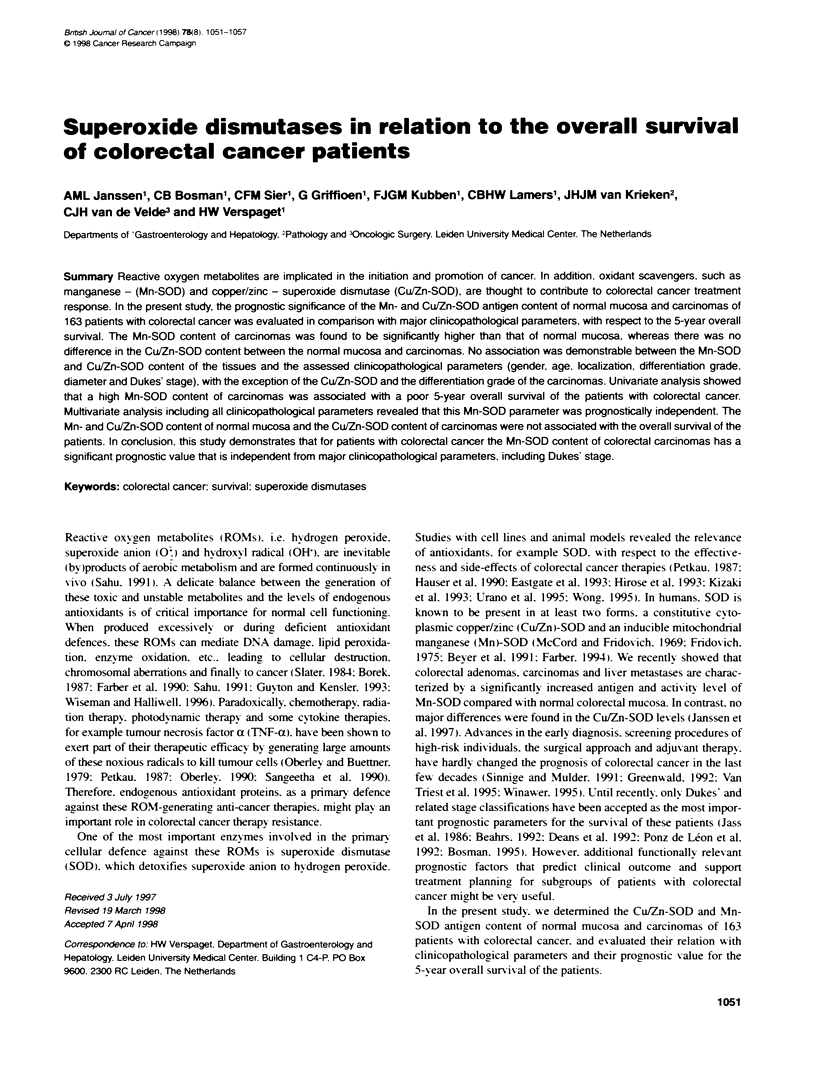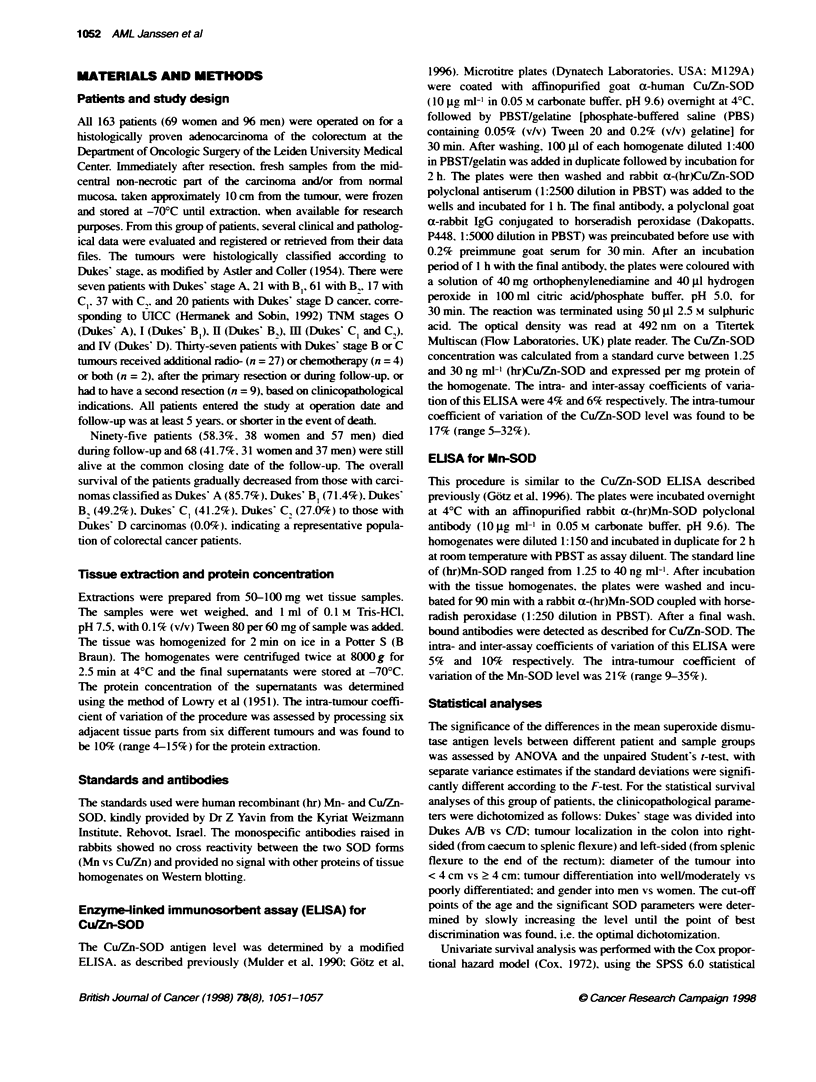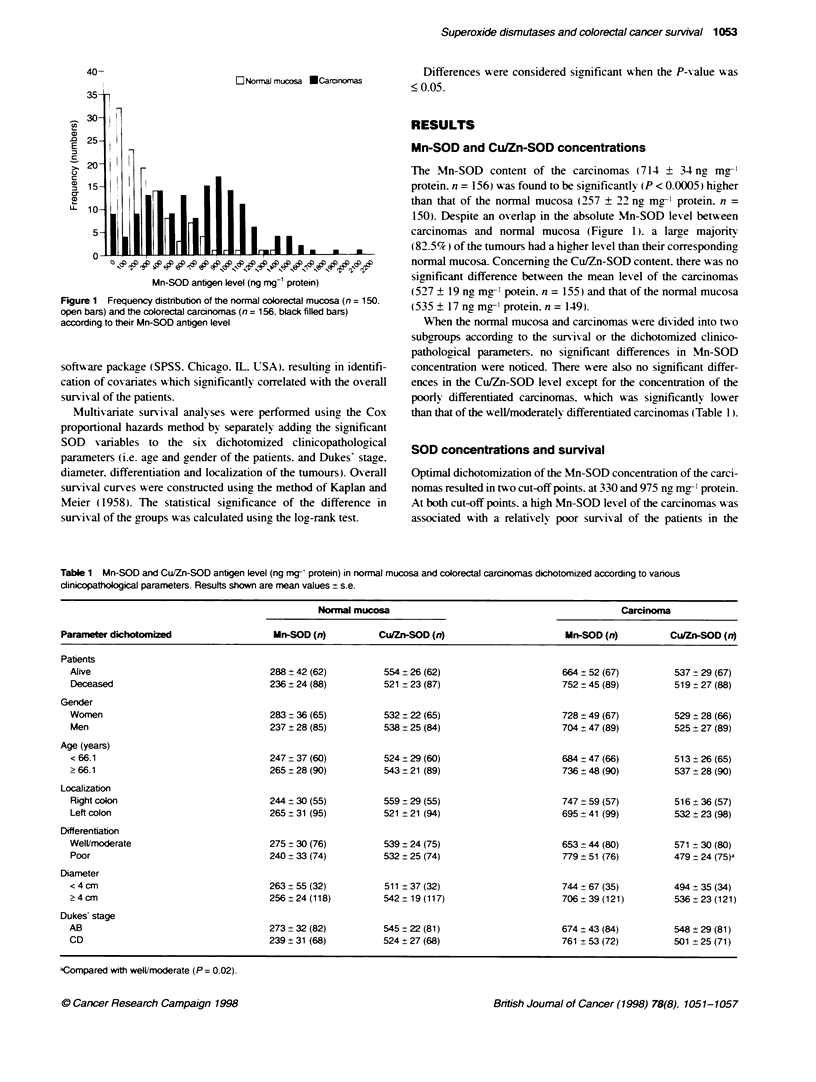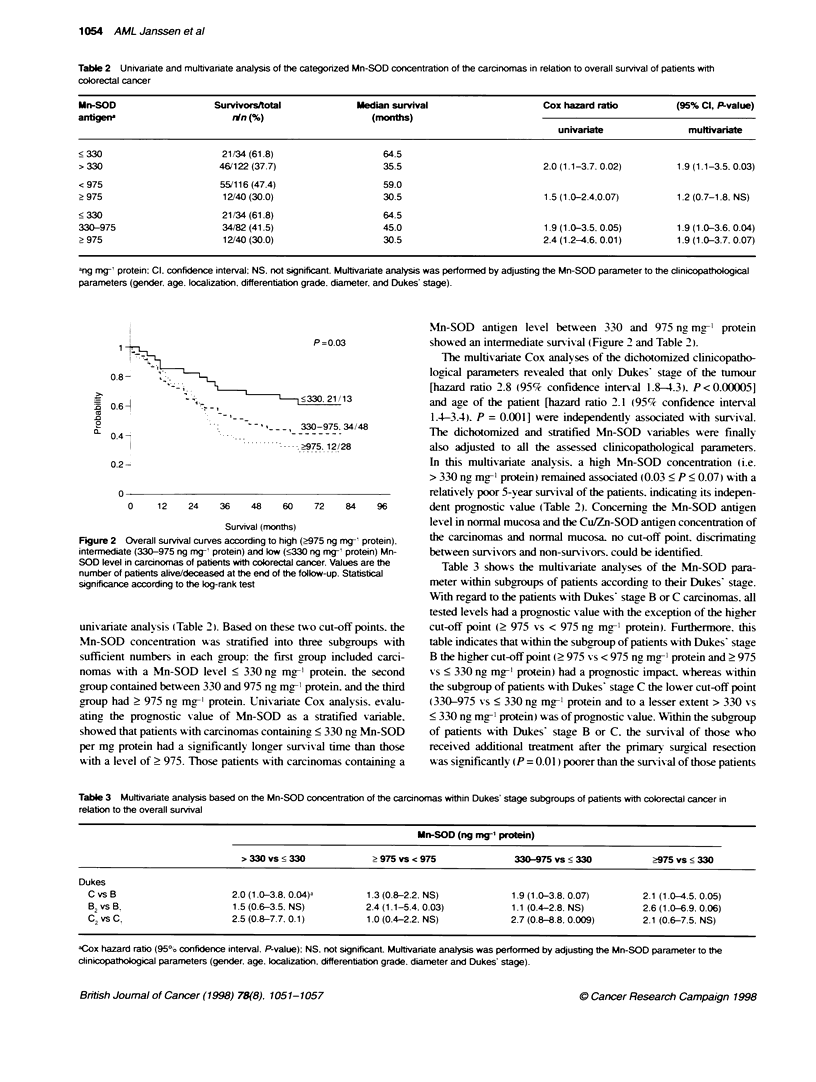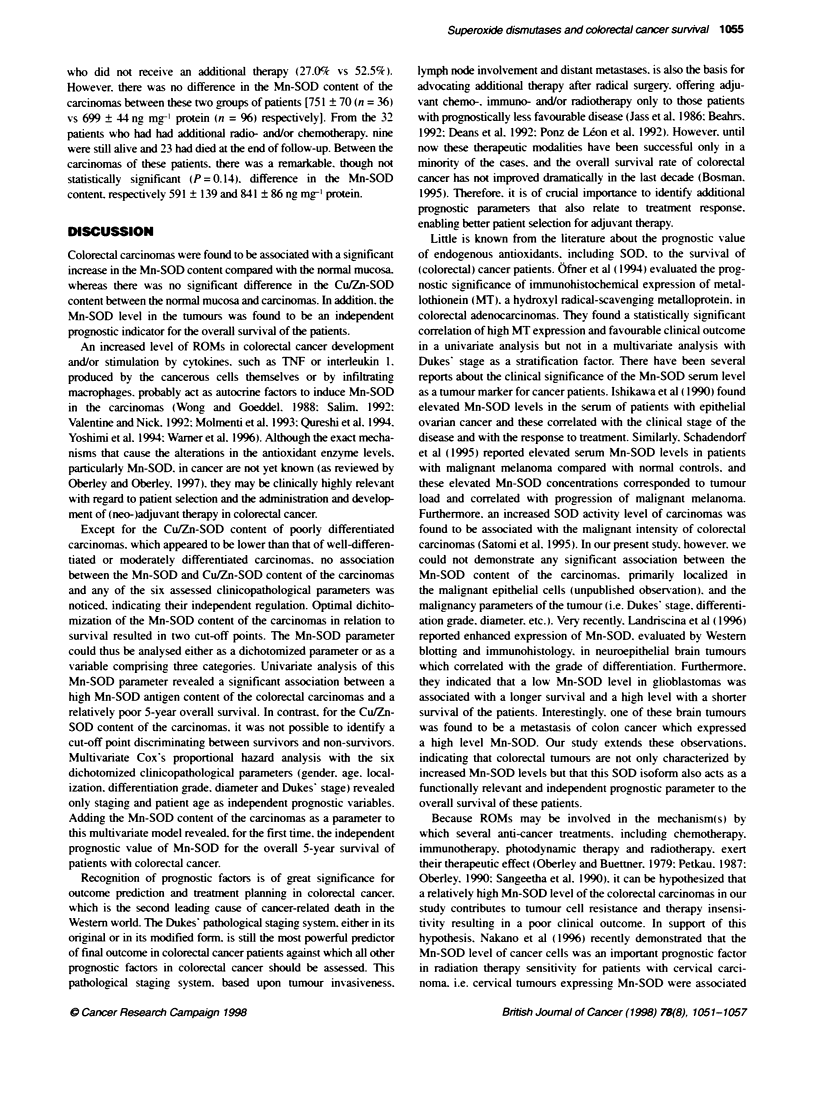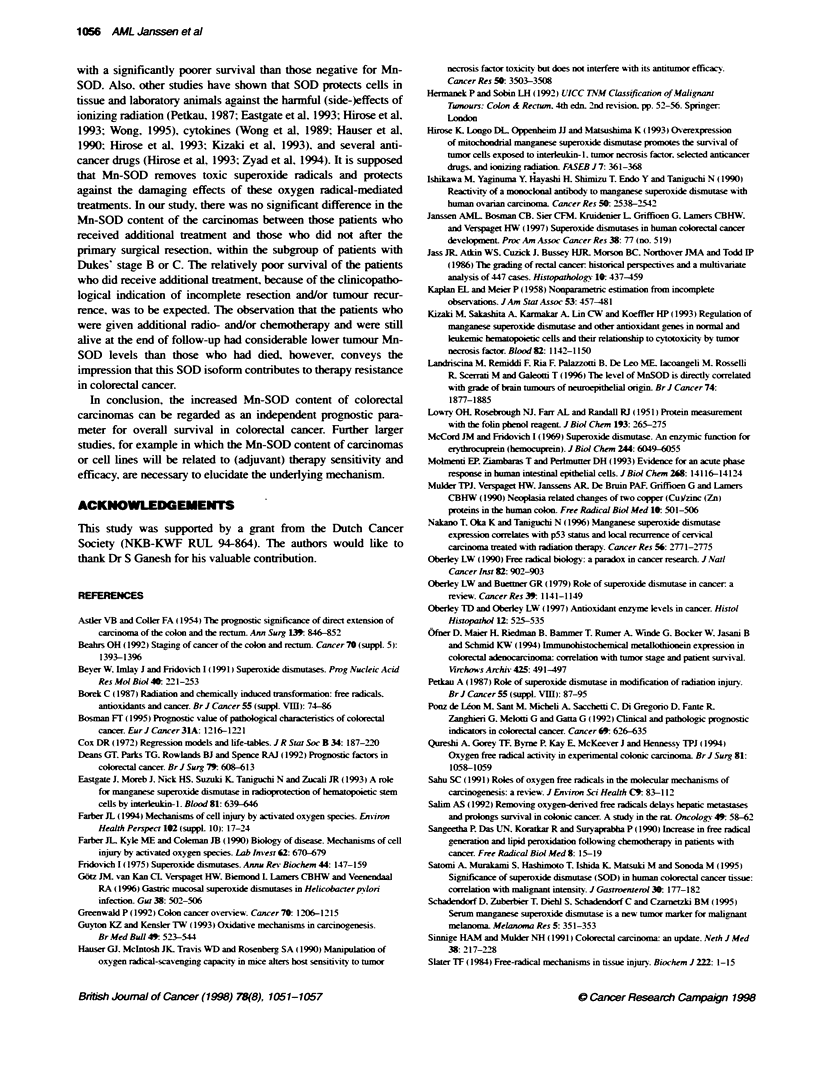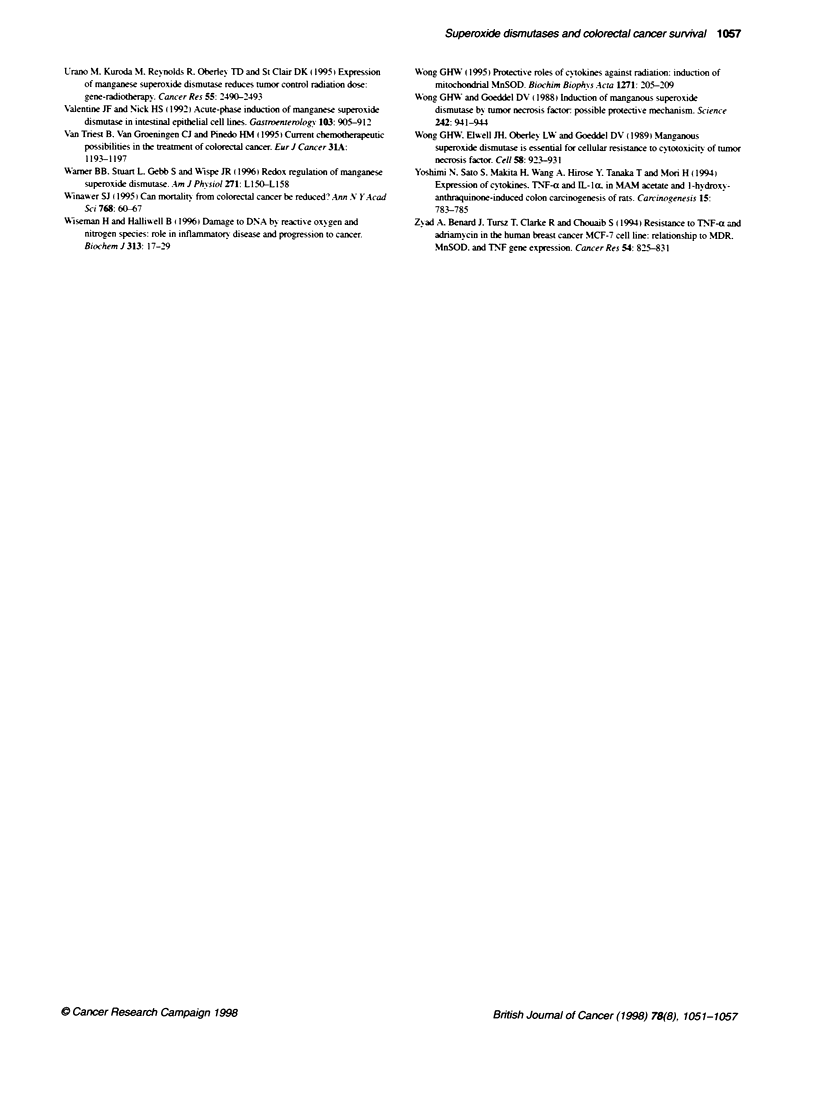# Superoxide dismutases in relation to the overall survival of colorectal cancer patients.

**DOI:** 10.1038/bjc.1998.626

**Published:** 1998-10

**Authors:** A. M. Janssen, C. B. Bosman, C. F. Sier, G. Griffioen, F. J. Kubben, C. B. Lamers, J. H. van Krieken, C. J. van de Velde, H. W. Verspaget

**Affiliations:** Department of Gastroenterology and Hepatology, Leiden University Medical Center, The Netherlands.

## Abstract

Reactive oxygen metabolites are implicated in the initiation and promotion of cancer. In addition, oxidant scavengers, such as manganese--(Mn-SOD) and copper/zinc--superoxide dismutase (Cu/Zn-SOD), are thought to contribute to colorectal cancer treatment response. In the present study, the prognostic significance of the Mn- and Cu/Zn-SOD antigen content of normal mucosa and carcinomas of 163 patients with colorectal cancer was evaluated in comparison with major clinicopathological parameters, with respect to the 5-year overall survival. The Mn-SOD content of carcinomas was found to be significantly higher than that of normal mucosa, whereas there was no difference in the Cu/Zn-SOD content between the normal mucosa and carcinomas. No association was demonstrable between the Mn-SOD and Cu/Zn-SOD content of the tissues and the assessed clinicopathological parameters (gender, age, localization, differentiation grade, diameter and Dukes' stage), with the exception of the Cu/Zn-SOD and the differentiation grade of the carcinomas. Univariate analysis showed that a high Mn-SOD content of carcinomas was associated with a poor 5-year overall survival of the patients with colorectal cancer. Multivariate analysis including all clinicopathological parameters revealed that this Mn-SOD parameter was prognostically independent. The Mn- and Cu/Zn-SOD content of normal mucosa and the Cu/Zn-SOD content of carcinomas were not associated with the overall survival of the patients. In conclusion, this study demonstrates that for patients with colorectal cancer the Mn-SOD content of colorectal carcinomas has a significant prognostic value that is independent from major clinicopathological parameters, including Dukes' stage.


British Journal of Cancer (1998) 78(8). 1051–1057
© 1998 Cancer Research Campaign

### Superoxide dismutases in relation to the overall survival of colorectal cancer patients

**AML Janssen[1], CB Bosman[1], CFM Sier[1], G Griffioen[1], FJGM Kubben[1], CBHW Lamers[1], JHJM van Krieken[2], CJH van de Velde[3] and HW Verspaget[1]**

Departments of [1]Gastroenterology and Hepatology, [2]Pathology and [3]Oncologic Surgery, Leiden University Medical Center, The Netherlands

**Summary** Reactive oxygen metabolites are implicated in the initiation and promotion of cancer. In addition, oxidant scavengers, such as manganese – (Mn-SOD) and copper/zinc – superoxide dismutase (Cu/Zn-SOD), are thought to contribute to colorectal cancer treatment response. In the present study, the prognostic significance of the Mn- and Cu/Zn-SOD antigen content of normal mucosa and carcinomas of 163 patients with colorectal cancer was evaluated in comparison with major clinicopathological parameters, with respect to the 5-year overall survival. The Mn-SOD content of carcinomas was found to be significantly higher than that of normal mucosa, whereas there was no difference in the Cu/Zn-SOD content between the normal mucosa and carcinomas. No association was demonstrable between the Mn-SOD and Cu/Zn-SOD content of the tissues and the assessed clinicopathological parameters (gender, age, localization, differentiation grade, diameter and Dukes' stage), with the exception of the Cu/Zn-SOD and the differentiation grade of the carcinomas. Univariate analysis showed that a high Mn-SOD content of carcinomas was associated with a poor 5-year overall survival of the patients with colorectal cancer. Multivariate analysis including all clinicopathological parameters revealed that this Mn-SOD parameter was prognostically independent. The Mn- and Cu/Zn-SOD content of normal mucosa and the Cu/Zn-SOD content of carcinomas were not associated with the overall survival of the patients. In conclusion, this study demonstrates that for patients with colorectal cancer the Mn-SOD content of colorectal carcinomas has a significant prognostic value that is independent from major clinicopathological parameters, including Dukes' stage.

**Keywords:** colorectal cancer; survival; superoxide dismutases

Reactive oxygen metabolites (ROMs), i.e. hydrogen peroxide, superoxide anion ($O_2^{\cdot-}$) and hydroxyl radical ($OH^{\cdot}$), are inevitable (by)products of aerobic metabolism and are formed continuously in vivo (Sahu, 1991). A delicate balance between the generation of these toxic and unstable metabolites and the levels of endogenous antioxidants is of critical importance for normal cell functioning. When produced excessively or during deficient antioxidant defences, these ROMs can mediate DNA damage, lipid peroxidation, enzyme oxidation, etc., leading to cellular destruction, chromosomal aberrations and finally to cancer (Slater, 1984; Borek, 1987; Farber et al, 1990; Sahu, 1991; Guyton and Kensler, 1993; Wiseman and Halliwell, 1996). Paradoxically, chemotherapy, radiation therapy, photodynamic therapy and some cytokine therapies, for example tumour necrosis factor α (TNF-α), have been shown to exert part of their therapeutic efficacy by generating large amounts of these noxious radicals to kill tumour cells (Oberley and Buettner, 1979; Petkau, 1987; Oberley, 1990; Sangeetha et al, 1990). Therefore, endogenous antioxidant proteins, as a primary defence against these ROM-generating anti-cancer therapies, might play an important role in colorectal cancer therapy resistance.

One of the most important enzymes involved in the primary cellular defence against these ROMs is superoxide dismutase (SOD), which detoxifies superoxide anion to hydrogen peroxide.

*Received 3 July 1997*
*Revised 19 March 1998*
*Accepted 7 April 1998*

*Correspondence to:* HW Verspaget, Department of Gastroenterology and Hepatology, Leiden University Medical Center, Building 1 C4-P, PO Box 9600, 2300 RC Leiden, The Netherlands

Studies with cell lines and animal models revealed the relevance of antioxidants, for example SOD, with respect to the effectiveness and side-effects of colorectal cancer therapies (Petkau, 1987; Hauser et al, 1990; Eastgate et al, 1993; Hirose et al, 1993; Kizaki et al, 1993; Urano et al, 1995; Wong, 1995). In humans, SOD is known to be present in at least two forms, a constitutive cytoplasmic copper/zinc (Cu/Zn)-SOD and an inducible mitochondrial manganese (Mn)-SOD (McCord and Fridovich, 1969; Fridovich, 1975; Beyer et al, 1991; Farber, 1994). We recently showed that colorectal adenomas, carcinomas and liver metastases are characterized by a significantly increased antigen and activity level of Mn-SOD compared with normal colorectal mucosa. In contrast, no major differences were found in the Cu/Zn-SOD levels (Janssen et al, 1997). Advances in the early diagnosis, screening procedures of high-risk individuals, the surgical approach and adjuvant therapy, have hardly changed the prognosis of colorectal cancer in the last few decades (Sinnige and Mulder, 1991; Greenwald, 1992; Van Triest et al, 1995; Winawer, 1995). Until recently, only Dukes' and related stage classifications have been accepted as the most important prognostic parameters for the survival of these patients (Jass et al, 1986; Beahrs, 1992; Deans et al, 1992; Ponz de Léon et al, 1992; Bosman, 1995). However, additional functionally relevant prognostic factors that predict clinical outcome and support treatment planning for subgroups of patients with colorectal cancer might be very useful.

In the present study, we determined the Cu/Zn-SOD and Mn-SOD antigen content of normal mucosa and carcinomas of 163 patients with colorectal cancer, and evaluated their relation with clinicopathological parameters and their prognostic value for the 5-year overall survival of the patients.

### MATERIALS AND METHODS

### Patients and study design

All 163 patients (69 women and 96 men) were operated on for a histologically proven adenocarcinoma of the colorectum at the Department of Oncologic Surgery of the Leiden University Medical Center. Immediately after resection, fresh samples from the mid-central non-necrotic part of the carcinoma and/or from normal mucosa, taken approximately 10 cm from the tumour, were frozen and stored at –70°C until extraction, when available for research purposes. From this group of patients, several clinical and pathological data were evaluated and registered or retrieved from their data files. The tumours were histologically classified according to Dukes' stage, as modified by Astler and Coller (1954). There were seven patients with Dukes' stage A, 21 with $B_1$, 61 with $B_2$, 17 with $C_1$, 37 with $C_2$, and 20 patients with Dukes' stage D cancer, corresponding to UICC (Hermanek and Sobin, 1992) TNM stages O (Dukes' A), I (Dukes' $B_1$), II (Dukes' $B_2$), III (Dukes' $C_1$ and $C_2$), and IV (Dukes' D). Thirty-seven patients with Dukes' stage B or C tumours received additional radio- ($n = 27$) or chemotherapy ($n = 4$) or both ($n = 2$), after the primary resection or during follow-up, or had to have a second resection ($n = 9$), based on clinicopathological indications. All patients entered the study at operation date and follow-up was at least 5 years, or shorter in the event of death.

Ninety-five patients (58.3%, 38 women and 57 men) died during follow-up and 68 (41.7%, 31 women and 37 men) were still alive at the common closing date of the follow-up. The overall survival of the patients gradually decreased from those with carcinomas classified as Dukes' A (85.7%), Dukes' $B_1$ (71.4%), Dukes' $B_2$ (49.2%), Dukes' $C_1$ (41.2%), Dukes' $C_2$ (27.0%) to those with Dukes' D carcinomas (0.0%), indicating a representative population of colorectal cancer patients.

### Tissue extraction and protein concentration

Extractions were prepared from 50–100 mg wet tissue samples. The samples were wet weighed, and 1 ml of 0.1 M Tris-HCl, pH 7.5, with 0.1% (v/v) Tween 80 per 60 mg of sample was added. The tissue was homogenized for 2 min on ice in a Potter S (B Braun). The homogenates were centrifuged twice at 8000 *g* for 2.5 min at 4°C and the final supernatants were stored at –70°C. The protein concentration of the supernatants was determined using the method of Lowry et al (1951). The intra-tumour coefficient of variation of the procedure was assessed by processing six adjacent tissue parts from six different tumours and was found to be 10% (range 4–15%) for the protein extraction.

### Standards and antibodies

The standards used were human recombinant (hr) Mn- and Cu/Zn-SOD, kindly provided by Dr Z Yavin from the Kyriat Weizmann Institute, Rehovot, Israel. The monospecific antibodies raised in rabbits showed no cross reactivity between the two SOD forms (Mn vs Cu/Zn) and provided no signal with other proteins of tissue homogenates on Western blotting.

### Enzyme-linked immunosorbent assay (ELISA) for Cu/Zn-SOD

The Cu/Zn-SOD antigen level was determined by a modified ELISA, as described previously (Mulder et al, 1990; Götz et al, 1996). Microtitre plates (Dynatech Laboratories, USA: M129A) were coated with affinopurified goat α-human Cu/Zn-SOD (10 μg $ml^{-1}$ in 0.05 M carbonate buffer, pH 9.6) overnight at 4°C, followed by PBST/gelatine [phosphate-buffered saline (PBS) containing 0.05% (v/v) Tween 20 and 0.2% (v/v) gelatine] for 30 min. After washing, 100 μl of each homogenate diluted 1:400 in PBST/gelatin was added in duplicate followed by incubation for 2 h. The plates were then washed and rabbit α-(hr)Cu/Zn-SOD polyclonal antiserum (1:2500 dilution in PBST) was added to the wells and incubated for 1 h. The final antibody, a polyclonal goat α-rabbit IgG conjugated to horseradish peroxidase (Dakopatts, P448, 1:5000 dilution in PBST) was preincubated before use with 0.2% preimmune goat serum for 30 min. After an incubation period of 1 h with the final antibody, the plates were coloured with a solution of 40 mg orthophenylenediamine and 40 μl hydrogen peroxide in 100 ml citric acid/phosphate buffer, pH 5.0, for 30 min. The reaction was terminated using 50 μl 2.5 M sulphuric acid. The optical density was read at 492 nm on a Titertek Multiscan (Flow Laboratories, UK) plate reader. The Cu/Zn-SOD concentration was calculated from a standard curve between 1.25 and 30 ng $ml^{-1}$ (hr)Cu/Zn-SOD and expressed per mg protein of the homogenate. The intra- and inter-assay coefficients of variation of this ELISA were 4% and 6% respectively. The intra-tumour coefficient of variation of the Cu/Zn-SOD level was found to be 17% (range 5–32%).

### ELISA for Mn-SOD

This procedure is similar to the Cu/Zn-SOD ELISA described previously (Götz et al, 1996). The plates were incubated overnight at 4°C with an affinopurified rabbit α-(hr)Mn-SOD polyclonal antibody (10 μg $ml^{-1}$ in 0.05 M carbonate buffer, pH 9.6). The homogenates were diluted 1:150 and incubated in duplicate for 2 h at room temperature with PBST as assay diluent. The standard line of (hr)Mn-SOD ranged from 1.25 to 40 ng $ml^{-1}$. After incubation with the tissue homogenates, the plates were washed and incubated for 90 min with a rabbit α-(hr)Mn-SOD coupled with horseradish peroxidase (1:250 dilution in PBST). After a final wash, bound antibodies were detected as described for Cu/Zn-SOD. The intra- and inter-assay coefficients of variation of this ELISA were 5% and 10% respectively. The intra-tumour coefficient of variation of the Mn-SOD level was 21% (range 9–35%).

### Statistical analyses

The significance of the differences in the mean superoxide dismutase antigen levels between different patient and sample groups was assessed by ANOVA and the unpaired Student's *t*-test, with separate variance estimates if the standard deviations were significantly different according to the *F*-test. For the statistical survival analyses of this group of patients, the clinicopathological parameters were dichotomized as follows: Dukes' stage was divided into Dukes A/B vs C/D; tumour localization in the colon into right-sided (from caecum to splenic flexure) and left-sided (from splenic flexure to the end of the rectum); diameter of the tumour into $< 4$ cm vs $\geq 4$ cm; tumour differentiation into well/moderately vs poorly differentiated; and gender into men vs women. The cut-off points of the age and the significant SOD parameters were determined by slowly increasing the level until the point of best discrimination was found, i.e. the optimal dichotomization.

Univariate survival analysis was performed with the Cox proportional hazard model (Cox, 1972), using the SPSS 6.0 statistical

© Cancer Research Campaign 1998

**Figure 1** Frequency distribution of the normal colorectal mucosa ($n$ = 150, open bars) and the colorectal carcinomas ($n$ = 156, black filled bars) according to their Mn-SOD antigen level software package (SPSS, Chicago, IL, USA), resulting in identification of covariates which significantly correlated with the overall survival of the patients.

Multivariate survival analyses were performed using the Cox proportional hazards method by separately adding the significant SOD variables to the six dichotomized clinicopathological parameters (i.e. age and gender of the patients, and Dukes' stage, diameter, differentiation and localization of the tumours). Overall survival curves were constructed using the method of Kaplan and Meier (1958). The statistical significance of the difference in survival of the groups was calculated using the log-rank test.

Differences were considered significant when the $P$-value was $\leq 0.05$.

### RESULTS

### Mn-SOD and Cu/Zn-SOD concentrations

The Mn-SOD content of the carcinomas (714 ± 34 ng mg$^{-1}$ protein, $n$ = 156) was found to be significantly ($P < 0.0005$) higher than that of the normal mucosa (257 ± 22 ng mg$^{-1}$ protein, $n$ = 150). Despite an overlap in the absolute Mn-SOD level between carcinomas and normal mucosa (Figure 1), a large majority (82.5%) of the tumours had a higher level than their corresponding normal mucosa. Concerning the Cu/Zn-SOD content, there was no significant difference between the mean level of the carcinomas (527 ± 19 ng mg$^{-1}$ potein, $n$ = 155) and that of the normal mucosa (535 ± 17 ng mg$^{-1}$ protein, $n$ = 149).

When the normal mucosa and carcinomas were divided into two subgroups according to the survival or the dichotomized clinicopathological parameters, no significant differences in Mn-SOD concentration were noticed. There were also no significant differences in the Cu/Zn-SOD level except for the concentration of the poorly differentiated carcinomas, which was significantly lower than that of the well/moderately differentiated carcinomas (Table 1).

### SOD concentrations and survival

Optimal dichotomization of the Mn-SOD concentration of the carcinomas resulted in two cut-off points, at 330 and 975 ng mg$^{-1}$ protein. At both cut-off points, a high Mn-SOD level of the carcinomas was associated with a relatively poor survival of the patients in the

**Table 1** Mn-SOD and Cu/Zn-SOD antigen level (ng mg$^{-1}$ protein) in normal mucosa and colorectal carcinomas dichotomized according to various clinicopathological parameters. Results shown are mean values ± s.e.

| | Normal mucosa | | Carcinoma | |
|---|---|---|---|---|
| **Parameter dichotomized** | **Mn-SOD ($n$)** | **Cu/Zn-SOD ($n$)** | **Mn-SOD ($n$)** | **Cu/Zn-SOD ($n$)** |
| Patients | | | | |
| Alive | 288 ± 42 (62) | 554 ± 26 (62) | 664 ± 52 (67) | 537 ± 29 (67) |
| Deceased | 236 ± 24 (88) | 521 ± 23 (87) | 752 ± 45 (89) | 519 ± 27 (88) |
| Gender | | | | |
| Women | 283 ± 36 (65) | 532 ± 22 (65) | 728 ± 49 (67) | 529 ± 28 (66) |
| Men | 237 ± 28 (85) | 538 ± 25 (84) | 704 ± 47 (89) | 525 ± 27 (89) |
| Age (years) | | | | |
| < 66.1 | 247 ± 37 (60) | 524 ± 29 (60) | 684 ± 47 (66) | 513 ± 26 (65) |
| ≥ 66.1 | 265 ± 28 (90) | 543 ± 21 (89) | 736 ± 48 (90) | 537 ± 28 (90) |
| Localization | | | | |
| Right colon | 244 ± 30 (55) | 559 ± 29 (55) | 747 ± 59 (57) | 516 ± 36 (57) |
| Left colon | 265 ± 31 (95) | 521 ± 21 (94) | 695 ± 41 (99) | 532 ± 23 (98) |
| Differentiation | | | | |
| Well/moderate | 275 ± 30 (76) | 539 ± 24 (75) | 653 ± 44 (80) | 571 ± 30 (80) |
| Poor | 240 ± 33 (74) | 532 ± 25 (74) | 779 ± 51 (76) | 479 ± 24 (75)[a] |
| Diameter | | | | |
| < 4 cm | 263 ± 55 (32) | 511 ± 37 (32) | 744 ± 67 (35) | 494 ± 35 (34) |
| ≥ 4 cm | 256 ± 24 (118) | 542 ± 19 (117) | 706 ± 39 (121) | 536 ± 23 (121) |
| Dukes' stage | | | | |
| AB | 273 ± 32 (82) | 545 ± 22 (81) | 674 ± 43 (84) | 548 ± 29 (81) |
| CD | 239 ± 31 (68) | 524 ± 27 (68) | 761 ± 53 (72) | 501 ± 25 (71) |

[a]Compared with well/moderate ($P$ = 0.02).

**Table 2** Univariate and multivariate analysis of the categorized Mn-SOD concentration of the carcinomas in relation to overall survival of patients with colorectal cancer

| Mn-SOD antigen[a] | Survivors/total n/n (%) | Median survival (months) | Cox hazard ratio univariate | (95% CI, P-value) multivariate |
|---|---|---|---|---|
| ≤ 330 | 21/34 (61.8) | 64.5 | | |
| > 330 | 46/122 (37.7) | 35.5 | 2.0 (1.1–3.7. 0.02) | 1.9 (1.1–3.5. 0.03) |
| < 975 | 55/116 (47.4) | 59.0 | | |
| ≥ 975 | 12/40 (30.0) | 30.5 | 1.5 (1.0–2.4.0.07) | 1.2 (0.7–1.8. NS) |
| ≤ 330 | 21/34 (61.8) | 64.5 | | |
| 330–975 | 34/82 (41.5) | 45.0 | 1.9 (1.0–3.5. 0.05) | 1.9 (1.0–3.6. 0.04) |
| ≥ 975 | 12/40 (30.0) | 30.5 | 2.4 (1.2–4.6. 0.01) | 1.9 (1.0–3.7. 0.07) |

[a]ng mg$^{-1}$ protein; CI, confidence interval; NS, not significant. Multivariate analysis was performed by adjusting the Mn-SOD parameter to the clinicopathological parameters (gender, age, localization, differentiation grade, diameter, and Dukes' stage).

**Figure 2** Overall survival curves according to high (≥975 ng mg$^{-1}$ protein), intermediate (330–975 ng mg$^{-1}$ protein) and low (≤330 ng mg$^{-1}$ protein) Mn-SOD level in carcinomas of patients with colorectal cancer. Values are the number of patients alive/deceased at the end of the follow-up. Statistical significance according to the log-rank test univariate analysis (Table 2). Based on these two cut-off points, the Mn-SOD concentration was stratified into three subgroups with sufficient numbers in each group: the first group included carcinomas with a Mn-SOD level ≤ 330 ng mg$^{-1}$ protein, the second group contained between 330 and 975 ng mg$^{-1}$ protein, and the third group had ≥ 975 ng mg$^{-1}$ protein. Univariate Cox analysis, evaluating the prognostic value of Mn-SOD as a stratified variable, showed that patients with carcinomas containing ≤ 330 ng Mn-SOD per mg protein had a significantly longer survival time than those with a level of ≥ 975. Those patients with carcinomas containing a Mn-SOD antigen level between 330 and 975 ng mg$^{-1}$ protein showed an intermediate survival (Figure 2 and Table 2).

The multivariate Cox analyses of the dichotomized clinicopathological parameters revealed that only Dukes' stage of the tumour [hazard ratio 2.8 (95% confidence interval 1.8–4.3), $P < 0.00005$] and age of the patient [hazard ratio 2.1 (95% confidence interval 1.4–3.4), $P = 0.001$] were independently associated with survival. The dichotomized and stratified Mn-SOD variables were finally also adjusted to all the assessed clinicopathological parameters. In this multivariate analysis, a high Mn-SOD concentration (i.e. > 330 ng mg$^{-1}$ protein) remained associated ($0.03 \leq P \leq 0.07$) with a relatively poor 5-year survival of the patients, indicating its independent prognostic value (Table 2). Concerning the Mn-SOD antigen level in normal mucosa and the Cu/Zn-SOD antigen concentration of the carcinomas and normal mucosa, no cut-off point, discrimating between survivors and non-survivors, could be identified.

Table 3 shows the multivariate analyses of the Mn-SOD parameter within subgroups of patients according to their Dukes' stage. With regard to the patients with Dukes' stage B or C carcinomas, all tested levels had a prognostic value with the exception of the higher cut-off point (≥ 975 vs < 975 ng mg$^{-1}$ protein). Furthermore, this table indicates that within the subgroup of patients with Dukes' stage B the higher cut-off point (≥ 975 vs < 975 ng mg$^{-1}$ protein and ≥ 975 vs ≤ 330 ng mg$^{-1}$ protein) had a prognostic impact, whereas within the subgroup of patients with Dukes' stage C the lower cut-off point (330–975 vs ≤ 330 ng mg$^{-1}$ protein and to a lesser extent > 330 vs ≤ 330 ng mg$^{-1}$ protein) was of prognostic value. Within the subgroup of patients with Dukes' stage B or C, the survival of those who received additional treatment after the primary surgical resection was significantly ($P = 0.01$) poorer than the survival of those patients

**Table 3** Multivariate analysis based on the Mn-SOD concentration of the carcinomas within Dukes' stage subgroups of patients with colorectal cancer in relation to the overall survival

| | Mn-SOD (ng mg$^{-1}$ protein) | | | |
|---|---|---|---|---|
| | > 330 vs ≤ 330 | ≥ 975 vs < 975 | 330–975 vs ≤ 330 | ≥975 vs ≤ 330 |
| Dukes | | | | |
| C vs B | 2.0 (1.0–3.8. 0.04)[a] | 1.3 (0.8–2.2. NS) | 1.9 (1.0–3.8. 0.07) | 2.1 (1.0–4.5. 0.05) |
| $B_2$ vs $B_1$ | 1.5 (0.6–3.5. NS) | 2.4 (1.1–5.4. 0.03) | 1.1 (0.4–2.8. NS) | 2.6 (1.0–6.9. 0.06) |
| $C_2$ vs $C_1$ | 2.5 (0.8–7.7. 0.1) | 1.0 (0.4–2.2. NS) | 2.7 (0.8–8.8. 0.009) | 2.1 (0.6–7.5. NS) |

[a]Cox hazard ratio (95% confidence interval, P-value); NS, not significant. Multivariate analysis was performed by adjusting the Mn-SOD parameter to the clinicopathological parameters (gender, age, localization, differentiation grade, diameter and Dukes' stage).

© Cancer Research Campaign 1998

who did not receive an additional therapy (27.0% vs 52.5%). However, there was no difference in the Mn-SOD content of the carcinomas between these two groups of patients [751 ± 70 ($n$ = 36) vs 699 ± 44 ng $mg^{-1}$ protein ($n$ = 96) respectively]. From the 32 patients who had had additional radio- and/or chemotherapy, nine were still alive and 23 had died at the end of follow-up. Between the carcinomas of these patients, there was a remarkable, though not statistically significant ($P$ = 0.14), difference in the Mn-SOD content, respectively 591 ± 139 and 841 ± 86 ng $mg^{-1}$ protein.

### DISCUSSION

Colorectal carcinomas were found to be associated with a significant increase in the Mn-SOD content compared with the normal mucosa, whereas there was no significant difference in the Cu/Zn-SOD content between the normal mucosa and carcinomas. In addition, the Mn-SOD level in the tumours was found to be an independent prognostic indicator for the overall survival of the patients.

An increased level of ROMs in colorectal cancer development and/or stimulation by cytokines, such as TNF or interleukin 1, produced by the cancerous cells themselves or by infiltrating macrophages, probably act as autocrine factors to induce Mn-SOD in the carcinomas (Wong and Goeddel, 1988; Salim, 1992; Valentine and Nick, 1992; Molmenti et al, 1993; Qureshi et al, 1994, Yoshimi et al, 1994; Warner et al, 1996). Although the exact mechanisms that cause the alterations in the antioxidant enzyme levels, particularly Mn-SOD, in cancer are not yet known (as reviewed by Oberley and Oberley, 1997), they may be clinically highly relevant with regard to patient selection and the administration and development of (neo-)adjuvant therapy in colorectal cancer.

Except for the Cu/Zn-SOD content of poorly differentiated carcinomas, which appeared to be lower than that of well-differentiated or moderately differentiated carcinomas, no association between the Mn-SOD and Cu/Zn-SOD content of the carcinomas and any of the six assessed clinicopathological parameters was noticed, indicating their independent regulation. Optimal dichitomization of the Mn-SOD content of the carcinomas in relation to survival resulted in two cut-off points. The Mn-SOD parameter could thus be analysed either as a dichotomized parameter or as a variable comprising three categories. Univariate analysis of this Mn-SOD parameter revealed a significant association between a high Mn-SOD antigen content of the colorectal carcinomas and a relatively poor 5-year overall survival. In contrast, for the Cu/Zn-SOD content of the carcinomas, it was not possible to identify a cut-off point discriminating between survivors and non-survivors. Multivariate Cox's proportional hazard analysis with the six dichotomized clinicopathological parameters (gender, age, localization, differentiation grade, diameter and Dukes' stage) revealed only staging and patient age as independent prognostic variables. Adding the Mn-SOD content of the carcinomas as a parameter to this multivariate model revealed, for the first time, the independent prognostic value of Mn-SOD for the overall 5-year survival of patients with colorectal cancer.

Recognition of prognostic factors is of great significance for outcome prediction and treatment planning in colorectal cancer, which is the second leading cause of cancer-related death in the Western world. The Dukes' pathological staging system, either in its original or in its modified form, is still the most powerful predictor of final outcome in colorectal cancer patients against which all other prognostic factors in colorectal cancer should be assessed. This pathological staging system, based upon tumour invasiveness, lymph node involvement and distant metastases, is also the basis for advocating additional therapy after radical surgery, offering adjuvant chemo-, immuno- and/or radiotherapy only to those patients with prognostically less favourable disease (Jass et al, 1986; Beahrs, 1992; Deans et al, 1992; Ponz de Léon et al, 1992). However, until now these therapeutic modalities have been successful only in a minority of the cases, and the overall survival rate of colorectal cancer has not improved dramatically in the last decade (Bosman, 1995). Therefore, it is of crucial importance to identify additional prognostic parameters that also relate to treatment response, enabling better patient selection for adjuvant therapy.

Little is known from the literature about the prognostic value of endogenous antioxidants, including SOD, to the survival of (colorectal) cancer patients. Öfner et al (1994) evaluated the prognostic significance of immunohistochemical expression of metallothionein (MT), a hydroxyl radical-scavenging metalloprotein, in colorectal adenocarcinomas. They found a statistically significant correlation of high MT expression and favourable clinical outcome in a univariate analysis but not in a multivariate analysis with Dukes' stage as a stratification factor. There have been several reports about the clinical significance of the Mn-SOD serum level as a tumour marker for cancer patients. Ishikawa et al (1990) found elevated Mn-SOD levels in the serum of patients with epithelial ovarian cancer and these correlated with the clinical stage of the disease and with the response to treatment. Similarly, Schadendorf et al (1995) reported elevated serum Mn-SOD levels in patients with malignant melanoma compared with normal controls, and these elevated Mn-SOD concentrations corresponded to tumour load and correlated with progression of malignant melanoma. Furthermore, an increased SOD activity level of carcinomas was found to be associated with the malignant intensity of colorectal carcinomas (Satomi et al, 1995). In our present study, however, we could not demonstrate any significant association between the Mn-SOD content of the carcinomas, primarily localized in the malignant epithelial cells (unpublished observation), and the malignancy parameters of the tumour (i.e. Dukes' stage, differentiation grade, diameter, etc.). Very recently, Landriscina et al (1996) reported enhanced expression of Mn-SOD, evaluated by Western blotting and immunohistology, in neuroepithelial brain tumours which correlated with the grade of differentiation. Furthermore, they indicated that a low Mn-SOD level in glioblastomas was associated with a longer survival and a high level with a shorter survival of the patients. Interestingly, one of these brain tumours was found to be a metastasis of colon cancer which expressed a high level Mn-SOD. Our study extends these observations, indicating that colorectal tumours are not only characterized by increased Mn-SOD levels but that this SOD isoform also acts as a functionally relevant and independent prognostic parameter to the overall survival of these patients.

Because ROMs may be involved in the mechanism(s) by which several anti-cancer treatments, including chemotherapy, immunotherapy, photodynamic therapy and radiotherapy, exert their therapeutic effect (Oberley and Buettner, 1979; Petkau, 1987; Oberley, 1990; Sangeetha et al, 1990), it can be hypothesized that a relatively high Mn-SOD level of the colorectal carcinomas in our study contributes to tumour cell resistance and therapy insensitivity resulting in a poor clinical outcome. In support of this hypothesis, Nakano et al (1996) recently demonstrated that the Mn-SOD level of cancer cells was an important prognostic factor in radiation therapy sensitivity for patients with cervical carcinoma, i.e. cervical tumours expressing Mn-SOD were associated

© Cancer Research Campaign 1998

with a significantly poorer survival than those negative for Mn-SOD. Also, other studies have shown that SOD protects cells in tissue and laboratory animals against the harmful (side-)effects of ionizing radiation (Petkau, 1987; Eastgate et al, 1993; Hirose et al, 1993; Wong, 1995), cytokines (Wong et al, 1989; Hauser et al, 1990; Hirose et al, 1993; Kizaki et al, 1993), and several anti-cancer drugs (Hirose et al, 1993; Zyad et al, 1994). It is supposed that Mn-SOD removes toxic superoxide radicals and protects against the damaging effects of these oxygen radical-mediated treatments. In our study, there was no significant difference in the Mn-SOD content of the carcinomas between those patients who received additional treatment and those who did not after the primary surgical resection, within the subgroup of patients with Dukes' stage B or C. The relatively poor survival of the patients who did receive additional treatment, because of the clinicopathological indication of incomplete resection and/or tumour recurrence, was to be expected. The observation that the patients who were given additional radio- and/or chemotherapy and were still alive at the end of follow-up had considerable lower tumour Mn-SOD levels than those who had died, however, conveys the impression that this SOD isoform contributes to therapy resistance in colorectal cancer.

In conclusion, the increased Mn-SOD content of colorectal carcinomas can be regarded as an independent prognostic parameter for overall survival in colorectal cancer. Further larger studies, for example in which the Mn-SOD content of carcinomas or cell lines will be related to (adjuvant) therapy sensitivity and efficacy, are necessary to elucidate the underlying mechanism.

### ACKNOWLEDGEMENTS

This study was supported by a grant from the Dutch Cancer Society (NKB-KWF RUL 94-864). The authors would like to thank Dr S Ganesh for his valuable contribution.

### REFERENCES

Astler VB and Coller FA (1954) The prognostic significance of direct extension of carcinoma of the colon and the rectum. *Ann Surg* **139**: 846–852

Beahrs OH (1992) Staging of cancer of the colon and rectum. *Cancer* **70** (suppl. 5): 1393–1396

Beyer W, Imlay J and Fridovich I (1991) Superoxide dismutases. *Prog Nucleic Acid Res Mol Biol* **40**: 221–253

Borek C (1987) Radiation and chemically induced transformation: free radicals, antioxidants and cancer. *Br J Cancer* **55** (suppl. VIII): 74–86

Bosman FT (1995) Prognostic value of pathological characteristics of colorectal cancer. *Eur J Cancer* **31A**: 1216–1221

Cox DR (1972) Regression models and life-tables. *J R Stat Soc* **B 34**: 187–220

Deans GT, Parks TG, Rowlands BJ and Spence RAJ (1992) Prognostic factors in colorectal cancer. *Br J Surg* **79**: 608–613

Eastgate J, Moreb J, Nick HS, Suzuki K, Taniguchi N and Zucali JR (1993) A role for manganese superoxide dismutase in radioprotection of hematopoietic stem cells by interleukin-1. *Blood* **81**: 639–646

Farber JL (1994) Mechanisms of cell injury by activated oxygen species. *Environ Health Perspect* **102** (suppl. 10): 17–24

Farber JL, Kyle ME and Coleman JB (1990) Biology of disease. Mechanisms of cell injury by activated oxygen species. *Lab Invest* **62**: 670–679

Fridovich I (1975) Superoxide dismutases. *Annu Rev Biochem* **44**: 147–159

Götz JM, van Kan CI, Verspaget HW, Biemond I, Lamers CBHW and Veenendaal RA (1996) Gastric mucosal superoxide dismutases in *Helicobacter pylori* infection. *Gut* **38**: 502–506

Greenwald P (1992) Colon cancer overview. *Cancer* **70**: 1206–1215

Guyton KZ and Kensler TW (1993) Oxidative mechanisms in carcinogenesis. *Br Med Bull* **49**: 523–544

Hauser GJ, McIntosh JK, Travis WD and Rosenberg SA (1990) Manipulation of oxygen radical-scavenging capacity in mice alters host sensitivity to tumor necrosis factor toxicity but does not interfere with its antitumor efficacy. *Cancer Res* **50**: 3503–3508

Hermanek P and Sobin LH (1992) *UICC TNM Classification of Malignant Tumours: Colon & Rectum*, 4th edn, 2nd revision, pp. 52–56. Springer: London

Hirose K, Longo DL, Oppenheim JJ and Matsushima K (1993) Overexpression of mitochondrial manganese superoxide dismutase promotes the survival of tumor cells exposed to interleukin-1, tumor necrosis factor, selected anticancer drugs, and ionizing radiation. *FASEB J* **7**: 361–368

Ishikawa M, Yaginuma Y, Hayashi H, Shimizu T, Endo Y and Taniguchi N (1990) Reactivity of a monoclonal antibody to manganese superoxide dismutase with human ovarian carcinoma. *Cancer Res* **50**: 2538–2542

Janssen AML, Bosman CB, Sier CFM, Kruidenier L, Griffioen G, Lamers CBHW, and Verspaget HW (1997) Superoxide dismutases in human colorectal cancer development. *Proc Am Assoc Cancer Res* **38**: 77 (no. 519)

Jass JR, Atkin WS, Cuzick J, Bussey HJR, Morson BC, Northover JMA and Todd IP (1986) The grading of rectal cancer: historical perspectives and a multivariate analysis of 447 cases. *Histopathology* **10**: 437–459

Kaplan EL and Meier P (1958) Nonparametric estimation from incomplete observations. *J Am Stat Assoc* **53**: 457–481

Kizaki M, Sakashita A, Karmakar A, Lin CW and Koeffler HP (1993) Regulation of manganese superoxide dismutase and other antioxidant genes in normal and leukemic hematopoietic cells and their relationship to cytotoxicity by tumor necrosis factor. *Blood* **82**: 1142–1150

Landriscina M, Remiddi F, Ria F, Palazzotti B, De Leo ME, Iacoangeli M, Rosselli R, Scerrati M and Galeotti T (1996) The level of MnSOD is directly correlated with grade of brain tumours of neuroepithelial origin. *Br J Cancer* **74**: 1877–1885

Lowry OH, Rosebrough NJ, Farr AL and Randall RJ (1951) Protein measurement with the folin phenol reagent. *J Biol Chem* **193**: 265–275

McCord JM and Fridovich I (1969) Superoxide dismutase. An enzymic function for erythrocuprein (hemocuprein). *J Biol Chem* **244**: 6049–6055

Molmenti EP, Ziambaras T and Perlmutter DH (1993) Evidence for an acute phase response in human intestinal epithelial cells. *J Biol Chem* **268**: 14116–14124

Mulder TPJ, Verspaget HW, Janssens AR, De Bruin PAF, Griffioen G and Lamers CBHW (1990) Neoplasia related changes of two copper (Cu)/zinc (Zn) proteins in the human colon. *Free Radical Biol Med* **10**: 501–506

Nakano T, Oka K and Taniguchi N (1996) Manganese superoxide dismutase expression correlates with p53 status and local recurrence of cervical carcinoma treated with radiation therapy. *Cancer Res* **56**: 2771–2775

Oberley LW (1990) Free radical biology: a paradox in cancer research. *J Natl Cancer Inst* **82**: 902–903

Oberley LW and Buettner GR (1979) Role of superoxide dismutase in cancer: a review. *Cancer Res* **39**: 1141–1149

Oberley TD and Oberley LW (1997) Antioxidant enzyme levels in cancer. *Histol Histopathol* **12**: 525–535

Öfner D, Maier H, Riedman B, Bammer T, Rumer A, Winde G, Bocker W, Jasani B and Schmid KW (1994) Immunohistochemical metallothionein expression in colorectal adenocarcinoma: correlation with tumor stage and patient survival. *Virchows Archiv* **425**: 491–497

Petkau A (1987) Role of superoxide dismutase in modification of radiation injury. *Br J Cancer* **55** (suppl. VIII): 87–95

Ponz de Léon M, Sant M, Micheli A, Sacchetti C, Di Gregorio D, Fante R, Zanghieri G, Melotti G and Gatta G (1992) Clinical and pathologic prognostic indicators in colorectal cancer. *Cancer* **69**: 626–635

Qureshi A, Gorey TF, Byrne P, Kay E, McKeever J and Hennessy TPJ (1994) Oxygen free radical activity in experimental colonic carcinoma. *Br J Surg* **81**: 1058–1059

Sahu SC (1991) Roles of oxygen free radicals in the molecular mechanisms of carcinogenesis: a review. *J Environ Sci Health* **C9**: 83–112

Salim AS (1992) Removing oxygen-derived free radicals delays hepatic metastases and prolongs survival in colonic cancer. A study in the rat. *Oncology* **49**: 58–62

Sangeetha P, Das UN, Koratkar R and Suryaprabha P (1990) Increase in free radical generation and lipid peroxidation following chemotherapy in patients with cancer. *Free Radical Biol Med* **8**: 15–19

Satomi A, Murakami S, Hashimoto T, Ishida K, Matsuki M and Sonoda M (1995) Significance of superoxide dismutase (SOD) in human colorectal cancer tissue: correlation with malignant intensity. *J Gastroenterol* **30**: 177–182

Schadendorf D, Zuberbier T, Diehl S, Schadendorf C and Czarnetzki BM (1995) Serum manganese superoxide dismutase is a new tumor marker for malignant melanoma. *Melanoma Res* **5**: 351–353

Sinnige HAM and Mulder NH (1991) Colorectal carcinoma: an update. *Neth J Med* **38**: 217–228

Slater TF (1984) Free-radical mechanisms in tissue injury. *Biochem J* **222**: 1–15

 © Cancer Research Campaign 1998

Urano M. Kuroda M. Reynolds R. Oberley TD and St Clair DK (1995) Expression of manganese superoxide dismutase reduces tumor control radiation dose: gene-radiotherapy. *Cancer Res* **55**: 2490–2493

Valentine JF and Nick HS (1992) Acute-phase induction of manganese superoxide dismutase in intestinal epithelial cell lines. *Gastroenterology* **103**: 905–912

Van Triest B. Van Groeningen CJ and Pinedo HM (1995) Current chemotherapeutic possibilities in the treatment of colorectal cancer. *Eur J Cancer* **31A**: 1193–1197

Warner BB. Stuart L. Gebb S and Wispe JR (1996) Redox regulation of manganese superoxide dismutase. *Am J Physiol* **271**: L150–L158

Winawer SJ (1995) Can mortality from colorectal cancer be reduced? *Ann N Y Acad Sci* **768**: 60–67

Wiseman H and Halliwell B (1996) Damage to DNA by reactive oxygen and nitrogen species: role in inflammatory disease and progression to cancer. *Biochem J* **313**: 17–29

Wong GHW (1995) Protective roles of cytokines against radiation: induction of mitochondrial MnSOD. *Biochim Biophys Acta* **1271**: 205–209

Wong GHW and Goeddel DV (1988) Induction of manganous superoxide dismutase by tumor necrosis factor: possible protective mechanism. *Science* **242**: 941–944

Wong GHW. Elwell JH. Oberley LW and Goeddel DV (1989) Manganous superoxide dismutase is essential for cellular resistance to cytotoxicity of tumor necrosis factor. *Cell* **58**: 923–931

Yoshimi N. Sato S. Makita H. Wang A. Hirose Y. Tanaka T and Mori H (1994) Expression of cytokines. TNF-α and IL-1α. in MAM acetate and 1-hydroxy-anthraquinone-induced colon carcinogenesis of rats. *Carcinogenesis* **15**: 783–785

Zyad A. Benard J. Tursz T. Clarke R and Chouaib S (1994) Resistance to TNF-α and adriamycin in the human breast cancer MCF-7 cell line: relationship to MDR. MnSOD. and TNF gene expression. *Cancer Res* **54**: 825–831

© Cancer Research Campaign 1998